# Corrigendum

**DOI:** 10.1111/bpa.13102

**Published:** 2022-06-26

In the article by Mawrin et al.,^1^ there were a couple of errors. The author, *Sina Neyazii,* was misspelt. It should have been *Sina Neyazi*. The article category should have been Research Article instead of Invited review.

The original article in the online version has been updated.

The authors apologize for the errors.
